# Conserved region of human TDP-43 is structurally similar to membrane binding protein FARP1 and protein chaperons BAG6 and CYP33

**DOI:** 10.17912/micropub.biology.001388

**Published:** 2024-11-07

**Authors:** Ljiljana Sjekloća, Emanuele Buratti

**Affiliations:** 1 Molecular Pathology, International Centre for Genetic Engineering and Biotechnology, Trieste, Italy

## Abstract

Transactive response DNA-binding protein of 43 KDa (TDP-43) is important for RNA metabolism in all animals and in humans is involved in neuromuscular diseases. Full-length TDP-43 is prone to oligomerization and misfolding what renders difficult its characterization. We report that TDP-43 domains are structurally similar to lipid binding protein FARP1 and protein chaperons BAG6 and CYP33. Sequence analysis suggests putative lipid binding sites throughout TDP-43 and in vitro thioflavin T fluorescence assays show that cholesterol and phosphatidylcholine affect fibrillation of recombinant TDP-43 fragments. Our findings suggest that TDP-43 can bind lipids directly and it may contribute to its own chaperoning.

**Figure 1. TDP-43 amino terminal domain NTD and RNA recognition motifs RRM are structurally similar to membrane-binding protein FARP1 and protein chaperons BAG6 and CYP33:**
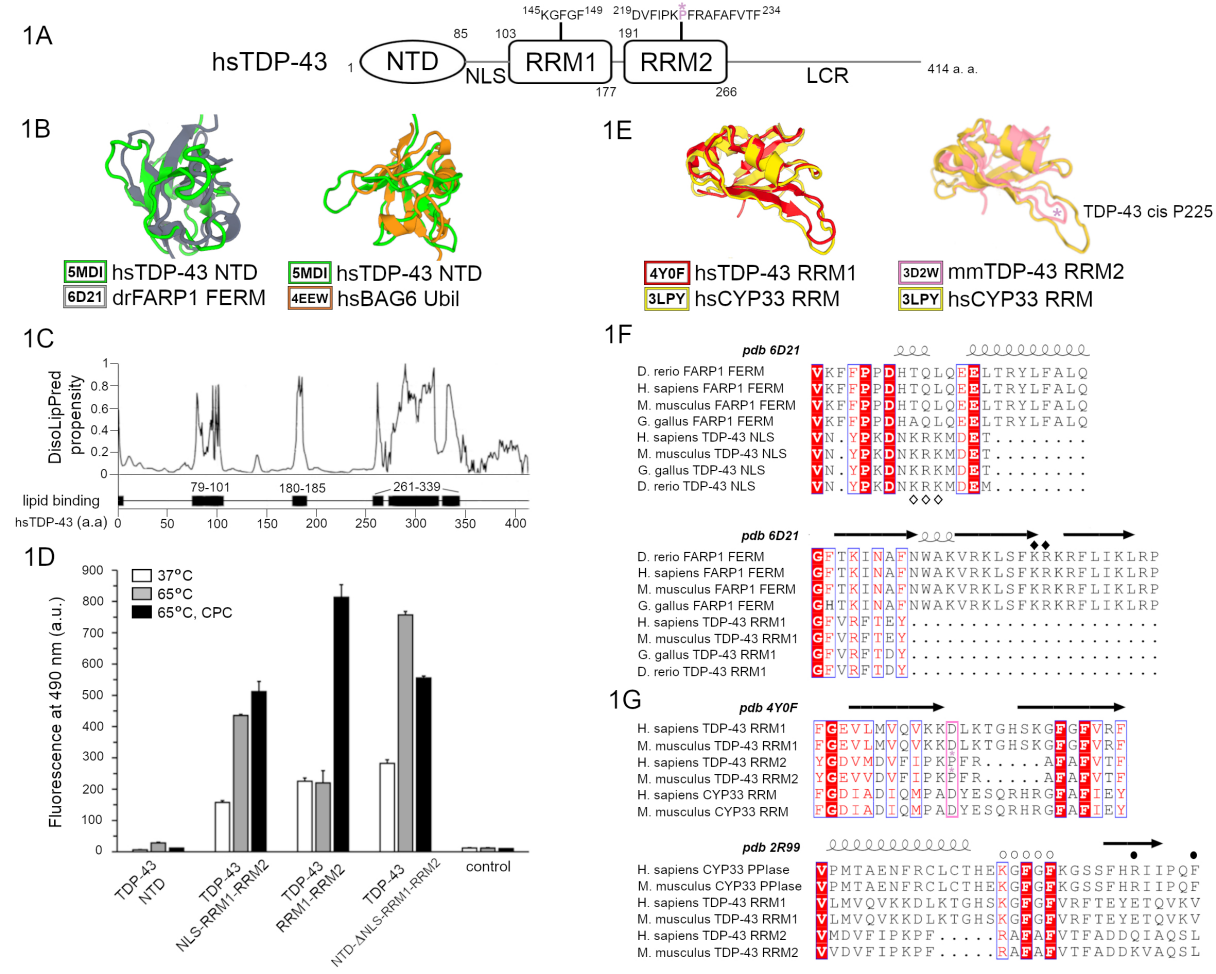
1A Schematic representation of human TDP-43 (hsTDP-43): NTD- amino terminal domain, NLS- nuclear localization signal, RRM-RNA recognition motif, LCR-low complexity region; PPIase-like sequence in RRM1 and putative aggregation and fibrillation-promoting sequence in RRM2 are put in evidence and cis P225 is shown in pink. 1B hsTDP-43 NTD domain superposition with FERM domain of zebra fish FARP1 and with ubiquitin-like domain of human BAG6 generated by DALI. 1C DisoLipPred propensity of hsTDP-43 residues to be in disordered lipid binding region plotted against TDP-43 sequence; predicted disordered lipid binding regions are indicated as black rectangles and numbered according to hsTDP-43 amino acid sequence. 1D Thioflavin T fluorescence of recombinantely produced hsTDP-43 constructs and control samples of thioflavin T incubated at physiological temperature 37°C or at 65°C in absence or presence of cholesterol (C) and phosphatidylcholine (PC); error bars denote standard errors of the mean from triplicate experiments. 1E hsTDP-43 RRM1 and mouse TDP-43 RRM2 motifs DALI-generated superposition to 3D model of hsCYP33 RRM domain; mmTDP43 RRM2 cis proline P225 is marked with the pink asterisk. 1F Clustal Omega-generated multiple sequences alignment of human, mouse, chicken and fish FARP1 and TDP-43, and ESPRIPT-generated rendering of secondary structure elements of zebra fish FERM domain relative to multiple sequences alignment information; white and black diamonds represent putative or experimentally confirmed lipid binding residues in TDP-43 and FARP1, respectively. 1G Multiple sequences alignment of human and mouse CYP33 RRM and PPIase domains, and human and mouse TDP-43 RRM1 and RRM2 motifs; ESPRIPT-generated rendering of secondary structure elements of hsTDP-43 RRM1 or hsCYP33 PPIase domain relative to multiple sequences alignment information; cis proline in TDP-43 RRM2 is denoted by the pink asterisk and this position is highlighted by pink rectangle in all aligned sequences; CYP33 residues involved in substrate binding are denoted by white spheres and some of hsCYP33 residues involved in peptidyl prolyl cis-trans isomerization are denoted by black spheres whilst the catalytic hsCYP33 S239 is not included because of the space limitations.

## Description

Transactive response DNA-binding protein of 43 kDa (TDP-43) is an essential DNA- and RNA-binding protein which in all animals regulates RNA metabolism, mitochondria biogenesis, chromatin remodelling and transcription (Freibaum et al., 2010; Berson et al., 2017; Neelagandan et al., 2019; Altman et al., 2021; Yu et al., 2021) . In humans TDP-43 is involved in neurodegenerative pathologies amyotrophic lateral sclerosis (ALS) and frontotemporal lobar dementia (FTLD) (Neumann et al., 2006) , and in degenerative myopathies sporadic inclusion bodies myositis (sIBM) (Cortese et al., 2014) and myoclonic epilepsy associated with ragged-red fibers (MERRF) (Mancuso et al., 2004; Mori et al., 2019) . In healthy individuals TDP-43 is predominantly a nuclear protein (Ayala et al., 2011; McGurk et al., 2018; Mann et al., 2019; Grese et al. 2021) though a small amount is present in cytosol, in rough endoplasmic reticulum and in mitochondria (Sasaki et al., 2010; Izumikawa et al., 2017; Duan et al., 2022) . Extracellularly, full length TDP-43 and its fragments have been observed as naked proteins or membrane-bound in extracellular vesicles in neuron-like cell cultures and in blood and cerebrospinal fluids of ALS and FTLD patients (Sackmann et al., 2020; Casarotto et al., 2022; Chattarjee et al., 2024) . Under cellular stress, nuclear TDP-43 translocates in cytoplasm where it may form condensates and, if the stress is prolonged, it becomes proteolytically fragmented, hyperphosphorylated and polyubiquitinated, and eventually aggregates in insoluble granules (reviewed by Tziortzouda et al. 2021). At molecular level, TDP-43 is a dimeric protein prone to oligomerization and it consists of conserved amino terminal domain NTD (1-77), a ~ 20 amino acid long linker containing nuclear export signal NES (82-98), two RNA recognition motifs (RRM1 104-176 and RRM2 192-266), and non-conserved, low complexity carboxyl terminal region (274-414) rich in glycine and glutamine/asparagine repeats ( Figure 1A ). All domains contribute to nucleic acids binding either by direct biding and/or by promoting protein dimerization which enhances RNA binding (Perez-Berlanga et al., 2023). TDP-43 is subjected to various post-translational modifications (Buratti, 2018) and our attention was attracted by peptidyl prolyl cis-trans isomerization in its RRM2 domain (Sjekloća and Buratti, 2024) as peptidyl prolyl cis-trans isomerization of phosphorylated Tau protein is a hallmark of an early pathogenic conformation in Alzheimer’s disease (Nakamura et al., 2013) .

Peptidyl prolyl isomerases (PPIases) assist cotranslational protein folding by favouring peptidyl prolyl cis-trans isomerization (reviewed by Schmid, 1993) and they are also involved in formation of higher order assemblies of intrinsically disordered proteins and in liquid-liquid phase separation of such proteins (Favretto et al., 2020; Babu et al., 2022) . Currently it is not known how relevant is peptidyl prolyl cis-trans isomerization for TDP-43 folding, stability and condensates formation, and if the cis proline P225 in TDP-43 RRM2 may be a recognition mark for TDP-43's interaction partners. In absence of high-resolution structures of the full length TDP-43 and of structures of its multidomain fragments, we compared available structures of human TDP-43 (hsTDP-43) NTD and RRM domains against the entire protein data bank PDB using DALI algorithms for protein structure comparison (Holm, 2020) . hsTDP-43 NTD is dimeric and it promotes full length hsTDP-43 oligomerization (Chang et al., 2012; Mompean et al., 2017) , and DALI comparison of its crystal structure, PDB 5MDI (Afroz et al., 2016) reveals it is similar to membrane-binding FERM domain of zebra fish synaptogenic protein FARP1, PDB 6D21 (Kuo et al., 2018; Z=7.2, rmsd= 2.6 Å; Figure 1B ) and to ubiquitin-like domain of human molecular chaperon BAG6, PDB 4EEW (Z=6.4, rmsd= 2.4 Å; Figure 1B ). FARP1 (FERM, RhoGEF and pleckstrin domain-containing protein 1/CDEP) is a neuronal cytoplasmic membrane-binding protein expressed in vertebrates, and in chicken and rats is a key regulator of embryonic growth of motor neurons subtypes and it contributes to synaptogenesis (Koyano et al., 1997; Zhuang et al., 2009; Cheadle and Biederer, 2014). Human BAG6 (BCL2-associated athanogene 6) is a large proline-rich multidomain cytoplasmic protein which prevents the aggregation of misfolded and hydrophobic patches-containing proteins maintaining them soluble and delivering them to endoplasmic reticulum and to proteasome (Wang et al., 2011) ; notably, human BAG6 recruits hsTDP-43 C-terminal proteolytic fragments CTF 219-414 and 247-414 to ubiquitin ligase RNF126 which directs them for degradation by proteasome (Kasu et al., 2022) .

Structural similarity of hsTDP-43 NTD to the membrane-binding FERM domain of FARP1 prompted us to analyze human TDP-43 sequence by bioinformatic tool DisoLipPred for prediction of disordered lipid binding residues (Katuwawala and Kurgan, 2021) and results suggest presence of such residues in hsTDP-43 NLS (amino acids 79-101), in linker which connects RRM1 and RRM2 (amino acids 180-185), and in LCR (amino acids 261-339) ( Figure 1C ). Lipid binding sites are generally buried in hydrophobic pockets and may not be solvent accessible in already folded protein. For that reason, we thermally perturbed recombinant hsTDP-43 samples NTD, NLS-RRM1-RRM2, RRM1-RRM2 and NTD-ΔNLS-RRM1-RRM2 (each at 50 μM concentration) by exposure to 65°C in presence of cholesterol and phosphatidylcholine, and determined if there was any effect on their *in vitro* aggregation properties based on amyloid-binding fluorescent dye thioflavin T (ThT) intensity.

In the absence of cholesterol and phosphatidylcholine, at physiological temperature 37°C, the hsTDP-43 NTD protein on its own cannot induce ThT fluorescence. However, at this temperature ThT fluorescence was increased when incubated with NTD-ΔNLS-RRM1-RRM2, NLS-RRM1-RRM2 or RRM1-RRM2 constructs ( Figure 1D, white bars). When TDP-43 samples were incubated at higher temperature (65°C), there was no ThT fluorescence in samples containing only NTD, while ThT fluorescence of NLS-RRM1-RRM2 and NTD-ΔNLS-RRM1-RRM2 doubled compared to the fluorescence of same constructs incubated at physiological temperature ( Figure 1D, grey bars). Finally, fluorescence of ThT incubated with RRM1-RRM2 at 65°C increased similarly to the fluorescence observed al physiological temperature ( Figure 1D, grey bars).

In the presence of cholesterol and phosphatidylcholine (50 μM and 250 μM, respectively- concentrations similar to those in cellular membranes; Chakravarthy et al., 1985; Bjorkhem and Meaney, 2004) and at 65°C, ThT fluorescence changed slightly for NLS-RRM1-RRM2 and NTD-ΔNLS-RRM1-RRM2 samples and to a much larger degree for RRM1-RRM2 ( Figure 1D, black bars) in comparison to samples incubated without any lipids ( Figure 1D, white and grey bars). Intriguingly, NTD could not induce ThT fluorescence also in the presence of lipids, suggesting that NTD does not bind thioflavin T under any examined conditions.

To better explain these data, we analyzed human TDP-43 sequence also by bioinformatic tools Aggrescan (Conchillo-Solé et al., 2007) and WALTZ (Louros et al., 2020) which both suggest presence of aggregation and fibrillation-promoting sequence in hsTDP-43 RRM2 (amino acids 219-234) which may explain why RRM2-containing TDP-43 fragments induce ThT fluorescence and NTD alone does not. Interestingly, this fibrillation site coincides with the amino terminal region of TDP-43 proteolytic fragment CTF 219-414 which *in vivo* forms intracellular insoluble aggregates and is found also in extracellular vesicles isolated from plasma of ALS patients (Zhang et al., 2007; Nonaka et al., 2009; Casarotto et al., 2022) . Multiple sequences alignment of FARP1 proteins and TDP-43 shows they both present a conserved KRK motif; in FERM domain of zebra fish FARP1 this sequence motif is exposed and in human FARP1 it includes K273 and R274 essential for *in vitro* binding to lipids (Kuo et al., 2018) . In hsTDP-43, the K ^82^ RK ^84^ sequence is located in the loop which connects the NTD and RRM domains ( Figure 1A, 1C and 1F) and acts as recognition sequence for importin α1/β (Ayala et al., 2008; Nishimura et al., 2010; Doll et al., 2022) .

Considering that TDP-43 RRM motifs are monomeric each on its own, and when in tandem (Kuo et al., 2009; Lukavsky et al., 2013) , for DALI comparison of RRM1 we used as query its crystal structure, PDB 4Y0F (Chiang et al., 2016) and NMR structure, PDB 4BS2 (Lukavsky et al., 2013; the RRM1 half). The results show they are both similar to the RRM domain of human peptidyl prolyl cis-trans isomerase PPIE/CYP33 (hereinafter CYP33) PDB 2KU7 and PDB 3MDF (Wang et al., 2011; Hom et al, 2010; Z=8.7, rmsd=1.6 Å; Z=11, rmsd=2.2 Å, similarity to 4Y0F and to 4BS2, respectively; Figure 1E L eft ). For DALI comparison of TDP-43 RRM2, we used as query mouse TDP-43 RRM2 crystal structure, PBD 3D2W (Kuo et al., 2009) and human TDP-43 RRM2 NMR structure, PDB 4BS2 (Lukavsky et al., 2013; the RRM2 half) and results show that also they are structurally similar to RRM of human CYP33, PDB 3LPY (Wang et al, 2011; Z=12.7, rmsd=1.4 Å, and Z=10.1, rmsd=1.8 Å, respectively for 3D2W and 4BS2; Figure 1E R ight). Nonetheless, CYP33 RRM and TDP-43 RRM domains differ in the loop which in hsTDP-43 RRM2 contains amino acids from F221 to F229 and comprises the cis P225; this loop is shorter in TDP-43 RRM2 than the topologically corresponding loops in CYP33 RRM and in TDP-43 RRM1, which don't contain any proline ( Figure 1E and 1G).

From a functional point of view, CYP33 is an essential human protein involved in splicing and chromatin remodelling, and it contains RRM and PPIase cyclophilin-type domain. CYP33 RRM domain binds preferentially single stranded polyA and polyU RNAs and RNA binding triggers the foldase activity of its PPIase domain (Wang et al., 2008; Loyd et al. 2021; Blatter et al., 2023) . We therefore aligned the full-length sequence of human CYP33, as well as the sequences of its separated RRM and PPIase domains, to the full-length sequence of human TDP-43 and sequences of its isolated domains ( Figure 1G ). In this comparison, we noticed that CYP33 sequence motif K ^180^ GFGV ^184^ involved in substrate binding and located in PPIase domain near the catalytic site is present also in TDP-43 RRM1 domain (hsTDP-43 K ^145^ GFGV ^149^ ), in the β3 strand that is directly involved in RNA binding (Lukavsky et al., 2013) . However, it should be considered that there are no nearby residues in TDP-43 sequence which correspond to catalytic arginine and serine typical of peptidyl prolyl cis-trans isomerases E, and in particular of CYP33 (Wang et al., 2005; Davies et al., 2010) ( Figure 1G ).

In summary, structural comparison of human TDP-43 domains revealed a similarity to membrane-binding protein FARP1 and to protein chaperons BAG6 and CYP33. Similarity of TDP-43 amino terminal domain to lipid-binding domain of FARP1 and the effects of cholesterol and phosphatidylcholine on *in vitro* fibrillation of recombinant TDP-43 constructs, suggest that TDP-43 can bind cellular membrane lipids and that lipids can affect TDP-43 propensity to aggregate. Notably, TDP-43 regulates splicing of proteins important for cholesterol biosynthesis (Egawa et al., 2022) and cholesterol metabolism is altered in ALS patients (Abdel-Khalik et al., 2017) , whilst phosphatidylcholine has neuroregenerative effects on cultured neural stem cells under inflammatory stress (Magaquian et al., 2021) . Direct contacts of TDP-43 and cell membrane lipids could therefore occur during TDP-43 intracellular and intercellular translocations and in extracellular vesicles isolated from the plasma of ALS and FTLD patients. Such interactions may influence cellular localization of TDP-43 as well as its packing into extracellular vesicles and may be relevant for cell-to-cell propagation of pathogenic TDP-43 in ALS and FTLD (Nonaka et al., 2013; Porta et al., 2018; Casarotto et al., 2022; Chattarjee et al., 2024) . In addition, the structural similarity of TDP-43 NTD and RRM motifs to protein chaperons BAG6 and CYP33, respectively, suggest that the TDP-43 protein might have an active, direct role in self-chaperoning. Indeed, multiple sequences alignment of TDP-43 and CYP33 reveals presence of a sequence motif in TDP-43 RRM1 which in CYP33 PPIase domain contributes to substrate binding. However, it is important to keep in mind that in TDP-43 there is no conservation of catalytic residues which are essential for CYP33 foldase activity. Hence, it is unlikely that TDP-43 may act as a peptidyl prolyl cis-trans isomerase. Nonetheless, in nature there are peptidyl prolyl cis-trans isomerases which act only as chaperons without acting as foldases. Hence, the similarity of TDP-43 to BAG6 and to CYP33 may suggest that TDP-43 could have chaperon activity which may be relevant for its aggregation or for organization of TDP-43-containing complexes and condensates. At the moment, it remains unknown which PPIase is responsible for peptidyl prolyl cis-trans isomerization in TDP-43 RRM2, although potential links between TDP-43 functions and PPIases cyclophilin A and PIN1 have been recently reported (Pasetto et al., 2021; Kato et al., 2022) . In theory, based on cyclophilin A’s and Pin1's substrate preferences, these PPIases may not be responsible for the peptidyl prolyl cis-trans isomerization of TDP-43 RRM2 P225. Nonetheless, other PPIases such as members of FKBP family may be responsible, and in particular those that are already linked to neurological diseases, for example FKBP12, FKBP51, or FKBP52 (Sugata et al., 2009, et al., 2011; Blair et al., 2013; Manabe et al., 2002) . Taken together, however, our results nonetheless support the hypothesis that further structural characterization of TDP-43 multidomain constructs and of the full length TDP-43 will presumably allow a thorough understanding of lipid binding and peptidyl prolyl cis-trans isomerization relevance for TDP-43 proteostasis.

## Methods

**Protein production**

Human TDP-43 (Uniprot Q13148) constructs were produced in *Escherichia coli* and purified as previously described (Sjekloca and Buratti, 2024). For all interaction essays we used only recombinant proteins without any tag and their purity and identity were checked by size exclusion chromatography (SEC), SDS-PAGE (NuPAGE Bis-Tris 4-12%, ThermoFisher) and mass spectrometry. Proteins were used in SEC buffer: 150 mM KCl, 20 mM NaCl, 1 mM MgCl _2_ , 5% glycerol, 50 mM Hepes-KOH pH 7.7, 2 mM 2- mercaptoethanol.

**Bioinformatic analysis**

Structure comparison was performed by DALI protein structure comparison server (Holm, 2020) ; crystal structure of TDP-43 NTD was used as query against entire PDB (Bernstein et al., 1977) ; crystal structures of RRM1 and RRM2 as well as NMR structure of tandem RRM1-RRM2 domains (used as halves) were used as queries. Multiple sequences alignment was performed by Clustal Omega (Madeira et al., 2024) and used together with PDB structures of TDP-43 NTD, RRM1 and CYP33 RRM and PPIase domains as input for ESPRIPT (Robert and Gouet, 2014) to render sequence similarities and secondary structure information; sequences used for alignment were retrieved from Uniprot protein data base: human TDP-43 (Q13148), mouse TDP-43 (Q921F2), chicken TDP-43 (Q5ZLN5), fish TDP-43(Q802C7), human FARP1 (Q9Y4F1), mouse FARP1 (F8VPU2), chicken FARP1 (F1P065), fish FARP1 (E9QIC8), human CYP33 (Q9UNP9) and mouse CYP33 (Q9QZH3). Human TDP-43 was analyzed by DisoLipPred (Katuwawala and Kurgan, 2021) , Aggrescan (Conchillo-Solé, 2007) and WALTZ (Louros et al., 2020) .

**Thioflavin T fluorescence measurements**

Thioflavin T (AbCam) was dissolved in SEC buffer at 1 mM concentration and filtered (0.22 μM cutoff). TDP-43 protein samples were at 50 μM concentration. Protein samples (150 μL) were incubated for 30 min at 37°C or 65°C, in a thermomixer set to 600 rpm (Eppendorf); 1 mM thioflavin T (1.5 μL) was added to final concentration 10 μM, and incubation was proceeded at 37°C or 65°C for another 30 min, at 600 rpm. Upon incubation, samples were briefly centrifuged at 100 rpm, delicately pipetted and transferred in an OptiPlate-96F microplate (PerkinElmer), and ThT fluorescence intensity was recorded in wavelength range 480-520 nm, using multimode plate reader (PerkinElmer EnVision 2104) with excitation filter of 450 nm. Cholesterol (Sigma) and phosphatidylcholine (Sigma) were dissolved separately to 100 mM in warm 100% methanol, mixed at 1:5 ratio in SEC buffer, vortexed for 60 sec and then used at final concentration of 50 μM and 250 μM, respectively, by adding their mixture to protein solution just before incubation at 65°C which lasted 30 minutes, after what 1 mM thioflavin T (1.5 μL) was added to final concentration 10 μM, and incubation was proceeded at 65°C for another 30 min, at 600 rpm. All experiments were repeated three times.

## References

[R1] Abdel-Khalik J, Yutuc E, Crick PJ, Gustafsson JÅ, Warner M, Roman G, Talbot K, Gray E, Griffiths WJ, Turner MR, Wang Y (2016). Defective cholesterol metabolism in amyotrophic lateral sclerosis.. J Lipid Res.

[R2] Afroz T, Hock EM, Ernst P, Foglieni C, Jambeau M, Gilhespy LAB, Laferriere F, Maniecka Z, Plückthun A, Mittl P, Paganetti P, Allain FHT, Polymenidou M (2017). Functional and dynamic polymerization of the ALS-linked protein TDP-43 antagonizes its pathologic aggregation.. Nat Commun.

[R3] Altman T, Ionescu A, Ibraheem A, Priesmann D, Gradus-Pery T, Farberov L, Alexandra G, Shelestovich N, Dafinca R, Shomron N, Rage F, Talbot K, Ward ME, Dori A, Krüger M, Perlson E (2021). Axonal TDP-43 condensates drive neuromuscular junction disruption through inhibition of local synthesis of nuclear encoded mitochondrial proteins.. Nat Commun.

[R4] Ayala YM, Zago P, D'Ambrogio A, Xu YF, Petrucelli L, Buratti E, Baralle FE (2008). Structural determinants of the cellular localization and shuttling of TDP-43.. J Cell Sci.

[R5] Ayala YM, De Conti L, Avendaño-Vázquez SE, Dhir A, Romano M, D'Ambrogio A, Tollervey J, Ule J, Baralle M, Buratti E, Baralle FE (2010). TDP-43 regulates its mRNA levels through a negative feedback loop.. EMBO J.

[R6] Babu M, Favretto F, Rankovic M, Zweckstetter M (2022). Peptidyl Prolyl Isomerase A Modulates the Liquid-Liquid Phase Separation of Proline-Rich IDPs.. J Am Chem Soc.

[R7] Bernstein FC, Koetzle TF, Williams GJ, Meyer EF Jr, Brice MD, Rodgers JR, Kennard O, Shimanouchi T, Tasumi M (1977). The Protein Data Bank: a computer-based archival file for macromolecular structures.. J Mol Biol.

[R8] Berson A, Sartoris A, Nativio R, Van Deerlin V, Toledo JB, Porta S, Liu S, Chung CY, Garcia BA, Lee VM, Trojanowski JQ, Johnson FB, Berger SL, Bonini NM (2017). TDP-43 Promotes Neurodegeneration by Impairing Chromatin Remodeling.. Curr Biol.

[R9] Björkhem I, Meaney S (2004). Brain cholesterol: long secret life behind a barrier.. Arterioscler Thromb Vasc Biol.

[R10] Blair LJ, Nordhues BA, Hill SE, Scaglione KM, O'Leary JC 3rd, Fontaine SN, Breydo L, Zhang B, Li P, Wang L, Cotman C, Paulson HL, Muschol M, Uversky VN, Klengel T, Binder EB, Kayed R, Golde TE, Berchtold N, Dickey CA (2013). Accelerated neurodegeneration through chaperone-mediated oligomerization of tau.. J Clin Invest.

[R11] Blatter M, Meylan C, Cléry A, Giambruno R, Nikolaev Y, Heidecker M, Solanki JA, Diaz MO, Gabellini D, Allain FH (2023). RNA binding induces an allosteric switch in Cyp33 to repress MLL1-mediated transcription.. Sci Adv.

[R12] Buratti E (2018). TDP-43 post-translational modifications in health and disease.. Expert Opin Ther Targets.

[R13] Casarotto E, Sproviero D, Corridori E, Gagliani MC, Cozzi M, Chierichetti M, Cristofani R, Ferrari V, Galbiati M, Mina F, Piccolella M, Rusmini P, Tedesco B, Gagliardi S, Cortese K, Cereda C, Poletti A, Crippa V (2022). Neurodegenerative Disease-Associated TDP-43 Fragments Are Extracellularly Secreted with CASA Complex Proteins.. Cells.

[R14] Chakravarthy BR, Spence MW, Clarke JT, Cook HW (1985). Rapid isolation of neuroblastoma plasma membranes on Percoll gradients. Characterization and lipid composition.. Biochim Biophys Acta.

[R15] Chang CK, Wu TH, Wu CY, Chiang MH, Toh EK, Hsu YC, Lin KF, Liao YH, Huang TH, Huang JJ (2012). The N-terminus of TDP-43 promotes its oligomerization and enhances DNA binding affinity.. Biochem Biophys Res Commun.

[R16] Chatterjee M, Özdemir S, Fritz C, Möbius W, Kleineidam L, Mandelkow E, Biernat J, Doğdu C, Peters O, Cosma NC, Wang X, Schneider LS, Priller J, Spruth E, Kühn AA, Krause P, Klockgether T, Vogt IR, Kimmich O, Spottke A, Hoffmann DC, Fliessbach K, Miklitz C, McCormick C, Weydt P, Falkenburger B, Brandt M, Guenther R, Dinter E, Wiltfang J, Hansen N, Bähr M, Zerr I, Flöel A, Nestor PJ, Düzel E, Glanz W, Incesoy E, Bürger K, Janowitz D, Perneczky R, Rauchmann BS, Hopfner F, Wagemann O, Levin J, Teipel S, Kilimann I, Goerss D, Prudlo J, Gasser T, Brockmann K, Mengel D, Zimmermann M, Synofzik M, Wilke C, Selma-González J, Turon-Sans J, Santos-Santos MA, Alcolea D, Rubio-Guerra S, Fortea J, Carbayo Á, Lleó A, Rojas-García R, Illán-Gala I, Wagner M, Frommann I, Roeske S, Bertram L, Heneka MT, Brosseron F, Ramirez A, Schmid M, Beschorner R, Halle A, Herms J, Neumann M, Barthélemy NR, Bateman RJ, Rizzu P, Heutink P, Dols-Icardo O, Höglinger G, Hermann A, Schneider A (2024). Plasma extracellular vesicle tau and TDP-43 as diagnostic biomarkers in FTD and ALS.. Nat Med.

[R17] Cheadle L, Biederer T (2014). Activity-dependent regulation of dendritic complexity by semaphorin 3A through Farp1.. J Neurosci.

[R18] Chiang CH, Grauffel C, Wu LS, Kuo PH, Doudeva LG, Lim C, Shen CK, Yuan HS (2016). Structural analysis of disease-related TDP-43 D169G mutation: linking enhanced stability and caspase cleavage efficiency to protein accumulation.. Sci Rep.

[R19] Conchillo-Solé O, de Groot NS, Avilés FX, Vendrell J, Daura X, Ventura S (2007). AGGRESCAN: a server for the prediction and evaluation of "hot spots" of aggregation in polypeptides.. BMC Bioinformatics.

[R20] Cortese A, Plagnol V, Brady S, Simone R, Lashley T, Acevedo-Arozena A, de Silva R, Greensmith L, Holton J, Hanna MG, Fisher EM, Fratta P (2013). Widespread RNA metabolism impairment in sporadic inclusion body myositis TDP43-proteinopathy.. Neurobiol Aging.

[R21] Davis TL, Walker JR, Campagna-Slater V, Finerty PJ, Paramanathan R, Bernstein G, MacKenzie F, Tempel W, Ouyang H, Lee WH, Eisenmesser EZ, Dhe-Paganon S (2010). Structural and biochemical characterization of the human cyclophilin family of peptidyl-prolyl isomerases.. PLoS Biol.

[R22] Deleersnijder A, Van Rompuy AS, Desender L, Pottel H, Buée L, Debyser Z, Baekelandt V, Gerard M (2011). Comparative analysis of different peptidyl-prolyl isomerases reveals FK506-binding protein 12 as the most potent enhancer of alpha-synuclein aggregation.. J Biol Chem.

[R23] Doll SG, Meshkin H, Bryer AJ, Li F, Ko YH, Lokareddy RK, Gillilan RE, Gupta K, Perilla JR, Cingolani G (2022). Recognition of the TDP-43 nuclear localization signal by importin α1/β.. Cell Rep.

[R24] Duan L, Zaepfel BL, Aksenova V, Dasso M, Rothstein JD, Kalab P, Hayes LR (2022). Nuclear RNA binding regulates TDP-43 nuclear localization and passive nuclear export.. Cell Rep.

[R25] Egawa N, Izumi Y, Suzuki H, Tsuge I, Fujita K, Shimano H, Izumikawa K, Takahashi N, Tsukita K, Enami T, Nakamura M, Watanabe A, Naitoh M, Suzuki S, Seki T, Kobayashi K, Toda T, Kaji R, Takahashi R, Inoue H (2022). TDP-43 regulates cholesterol biosynthesis by inhibiting sterol regulatory element-binding protein 2.. Sci Rep.

[R26] Favretto F, Flores D, Baker JD, Strohäker T, Andreas LB, Blair LJ, Becker S, Zweckstetter M (2020). Catalysis of proline isomerization and molecular chaperone activity in a tug-of-war.. Nat Commun.

[R27] Freibaum BD, Chitta RK, High AA, Taylor JP (2010). Global analysis of TDP-43 interacting proteins reveals strong association with RNA splicing and translation machinery.. J Proteome Res.

[R28] Grese ZR, Bastos AC, Mamede LD, French RL, Miller TM, Ayala YM (2021). Specific RNA interactions promote TDP-43 multivalent phase separation and maintain liquid properties.. EMBO Rep.

[R29] Holm L (2019). DALI and the persistence of protein shape.. Protein Sci.

[R30] Hom RA, Chang PY, Roy S, Musselman CA, Glass KC, Selezneva AI, Gozani O, Ismagilov RF, Cleary ML, Kutateladze TG (2010). Molecular mechanism of MLL PHD3 and RNA recognition by the Cyp33 RRM domain.. J Mol Biol.

[R31] Izumikawa K, Nobe Y, Yoshikawa H, Ishikawa H, Miura Y, Nakayama H, Nonaka T, Hasegawa M, Egawa N, Inoue H, Nishikawa K, Yamano K, Simpson RJ, Taoka M, Yamauchi Y, Isobe T, Takahashi N (2017). TDP-43 stabilises the processing intermediates of mitochondrial transcripts.. Sci Rep.

[R32] Kasu YAT, Arva A, Johnson J, Sajan C, Manzano J, Hennes A, Haynes J, Brower CS (2022). BAG6 prevents the aggregation of neurodegeneration-associated fragments of TDP43.. iScience.

[R33] Katuwawala A, Zhao B, Kurgan L (2021). DisoLipPred: accurate prediction of disordered lipid-binding residues in protein sequences with deep recurrent networks and transfer learning.. Bioinformatics.

[R34] Kuo PH, Doudeva LG, Wang YT, Shen CK, Yuan HS (2009). Structural insights into TDP-43 in nucleic-acid binding and domain interactions.. Nucleic Acids Res.

[R35] Kuo YC, He X, Coleman AJ, Chen YJ, Dasari P, Liou J, Biederer T, Zhang X (2018). Structural analyses of FERM domain-mediated membrane localization of FARP1.. Sci Rep.

[R36] Louros N, Konstantoulea K, De Vleeschouwer M, Ramakers M, Schymkowitz J, Rousseau F (2020). WALTZ-DB 2.0: an updated database containing structural information of experimentally determined amyloid-forming peptides.. Nucleic Acids Res.

[R37] Lukavsky PJ, Daujotyte D, Tollervey JR, Ule J, Stuani C, Buratti E, Baralle FE, Damberger FF, Allain FH (2013). Molecular basis of UG-rich RNA recognition by the human splicing factor TDP-43.. Nat Struct Mol Biol.

[R38] Madeira F, Madhusoodanan N, Lee J, Eusebi A, Niewielska A, Tivey ARN, Lopez R, Butcher S (2024). The EMBL-EBI Job Dispatcher sequence analysis tools framework in 2024.. Nucleic Acids Res.

[R39] Magaquian D, Delgado Ocaña S, Perez C, Banchio C (2021). Phosphatidylcholine&nbsp;restores neuronal plasticity&nbsp;of neural stem cells under inflammatory stress.. Sci Rep.

[R40] Mancuso M, Filosto M, Mootha VK, Rocchi A, Pistolesi S, Murri L, DiMauro S, Siciliano G (2004). A novel mitochondrial tRNAPhe mutation causes MERRF syndrome.. Neurology.

[R41] Mann JR, Gleixner AM, Mauna JC, Gomes E, DeChellis-Marks MR, Needham PG, Copley KE, Hurtle B, Portz B, Pyles NJ, Guo L, Calder CB, Wills ZP, Pandey UB, Kofler JK, Brodsky JL, Thathiah A, Shorter J, Donnelly CJ (2019). RNA Binding Antagonizes Neurotoxic Phase Transitions of TDP-43.. Neuron.

[R42] McGurk L, Gomes E, Guo L, Mojsilovic-Petrovic J, Tran V, Kalb RG, Shorter J, Bonini NM (2018). Poly(ADP-Ribose) Prevents Pathological Phase Separation of TDP-43 by Promoting Liquid Demixing and Stress Granule Localization.. Mol Cell.

[R43] Mompeán M, Romano V, Pantoja-Uceda D, Stuani C, Baralle FE, Buratti E, Laurents DV (2017). Point mutations in the N-terminal domain of transactive response DNA-binding protein 43 kDa (TDP-43) compromise its stability, dimerization, and functions.. J Biol Chem.

[R44] Mori F, Tada M, Kon T, Miki Y, Tanji K, Kurotaki H, Tomiyama M, Ishihara T, Onodera O, Kakita A, Wakabayashi K (2019). Phosphorylated TDP-43 aggregates in skeletal and cardiac muscle are a marker of myogenic degeneration in amyotrophic lateral sclerosis and various conditions.. Acta Neuropathol Commun.

[R45] Nakamura K, Zhen Zhou X, Ping Lu K (2012). Cis phosphorylated tau as the earliest detectable pathogenic conformation in Alzheimer disease, offering novel diagnostic and therapeutic strategies.. Prion.

[R46] Neelagandan N, Gonnella G, Dang S, Janiesch PC, Miller KK, Küchler K, Marques RF, Indenbirken D, Alawi M, Grundhoff A, Kurtz S, Duncan KE (2019). TDP-43 enhances translation of specific mRNAs linked to neurodegenerative disease.. Nucleic Acids Res.

[R47] Neumann M, Sampathu DM, Kwong LK, Truax AC, Micsenyi MC, Chou TT, Bruce J, Schuck T, Grossman M, Clark CM, McCluskey LF, Miller BL, Masliah E, Mackenzie IR, Feldman H, Feiden W, Kretzschmar HA, Trojanowski JQ, Lee VM (2006). Ubiquitinated TDP-43 in frontotemporal lobar degeneration and amyotrophic lateral sclerosis.. Science.

[R48] Nishimura AL, Zupunski V, Troakes C, Kathe C, Fratta P, Howell M, Gallo JM, Hortobágyi T, Shaw CE, Rogelj B (2010). Nuclear import impairment causes cytoplasmic trans-activation response DNA-binding protein accumulation and is associated with frontotemporal lobar degeneration.. Brain.

[R49] Nonaka T, Kametani F, Arai T, Akiyama H, Hasegawa M (2009). Truncation and pathogenic mutations facilitate the formation of intracellular aggregates of TDP-43.. Hum Mol Genet.

[R50] Nonaka T, Masuda-Suzukake M, Arai T, Hasegawa Y, Akatsu H, Obi T, Yoshida M, Murayama S, Mann DM, Akiyama H, Hasegawa M (2013). Prion-like properties of pathological TDP-43 aggregates from diseased brains.. Cell Rep.

[R51] Pasetto L, Grassano M, Pozzi S, Luotti S, Sammali E, Migazzi A, Basso M, Spagnolli G, Biasini E, Micotti E, Cerovic M, Carli M, Forloni G, De Marco G, Manera U, Moglia C, Mora G, Traynor BJ, Chiò A, Calvo A, Bonetto V (2021). Defective cyclophilin A induces TDP-43 proteinopathy: implications for amyotrophic lateral sclerosis and frontotemporal dementia.. Brain.

[R52] Pérez-Berlanga M, Wiersma VI, Zbinden A, De Vos L, Wagner U, Foglieni C, Mallona I, Betz KM, Cléry A, Weber J, Guo Z, Rigort R, de Rossi P, Manglunia R, Tantardini E, Sahadevan S, Stach O, Hruska-Plochan M, Allain FH, Paganetti P, Polymenidou M (2023). Loss of TDP-43 oligomerization or RNA binding elicits distinct aggregation patterns.. EMBO J.

[R53] Porta S, Xu Y, Restrepo CR, Kwong LK, Zhang B, Brown HJ, Lee EB, Trojanowski JQ, Lee VM (2018). Patient-derived frontotemporal lobar degeneration brain extracts induce formation and spreading of TDP-43 pathology in vivo.. Nat Commun.

[R54] Robert X, Gouet P (2014). Deciphering key features in protein structures with the new ENDscript server.. Nucleic Acids Res.

[R55] Sackmann C, Sackmann V, Hallbeck M (2020). TDP-43 Is Efficiently Transferred Between Neuron-Like Cells in a Manner Enhanced by Preservation of Its N-Terminus but Independent of Extracellular Vesicles.. Front Neurosci.

[R56] Sasaki S, Takeda T, Shibata N, Kobayashi M (2010). Alterations in subcellular localization of TDP-43 immunoreactivity in the anterior horns in sporadic amyotrophic lateral sclerosis.. Neurosci Lett.

[R57] Schmid F X (1993). Prolyl Isomerase: Enzymatic Catalysis of Slow Protein-Folding Reactions. Annual Review of Biophysics and Biomolecular Structure.

[R58] Sjekloća L, Buratti E (2024). tRNA (Arg) binds in vitro TDP-43 RNA recognition motifs and ligand of Ate1 protein LIAT1.. MicroPubl Biol.

[R59] Sugata H, Matsuo K, Nakagawa T, Takahashi M, Mukai H, Ono Y, Maeda K, Akiyama H, Kawamata T (2009). A peptidyl-prolyl isomerase, FKBP12, accumulates in Alzheimer neurofibrillary tangles.. Neurosci Lett.

[R60] Tziortzouda P, Van Den Bosch L, Hirth F (2021). Triad of TDP43 control in neurodegeneration: autoregulation, localization and aggregation.. Nat Rev Neurosci.

[R61] Wang Q, Liu Y, Soetandyo N, Baek K, Hegde R, Ye Y (2011). A ubiquitin ligase-associated chaperone holdase maintains polypeptides in soluble states for proteasome degradation.. Mol Cell.

[R62] Wang Y, Han R, Zhang W, Yuan Y, Zhang X, Long Y, Mi H (2008). Human CyP33 binds specifically to mRNA and binding stimulates PPIase activity of hCyP33.. FEBS Lett.

[R63] Wang Z, Song J, Milne TA, Wang GG, Li H, Allis CD, Patel DJ (2010). Pro isomerization in MLL1 PHD3-bromo cassette connects H3K4me readout to CyP33 and HDAC-mediated repression.. Cell.

[R64] Xiao Y, Wang SK, Zhang Y, Rostami A, Kenkare A, Casella G, Yuan ZQ, Li X (2021). Role of extracellular vesicles in neurodegenerative diseases.. Prog Neurobiol.

[R65] Yu H, Lu S, Gasior K, Singh D, Vazquez-Sanchez S, Tapia O, Toprani D, Beccari MS, Yates JR 3rd, Da Cruz S, Newby JM, Lafarga M, Gladfelter AS, Villa E, Cleveland DW (2020). HSP70 chaperones RNA-free TDP-43 into anisotropic intranuclear liquid spherical shells.. Science.

[R66] Zhang YJ, Xu YF, Dickey CA, Buratti E, Baralle F, Bailey R, Pickering-Brown S, Dickson D, Petrucelli L (2007). Progranulin mediates caspase-dependent cleavage of TAR DNA binding protein-43.. J Neurosci.

[R67] Zhuang B, Su YS, Sockanathan S (2009). FARP1 promotes the dendritic growth of spinal motor neuron subtypes through transmembrane Semaphorin6A and PlexinA4 signaling.. Neuron.

